# Paeoniflorin Ameliorates Hyperprolactinemia-Induced Inhibition of Osteoblastogenesis by Suppressing the NF-*κ*B Signaling Pathway

**DOI:** 10.1155/2022/4572033

**Published:** 2022-04-15

**Authors:** Xiaohong Sun, Keda Zhu, Chengcheng Feng, Jie Zhu, Shuangshuang Chen, Wenkai Tang, Zhifang Wang, Long Xiao, Hong Li, Dechun Geng, Zhirong Wang

**Affiliations:** ^1^Center Laboratory, Zhangjiagang TCM Hospital Affiliated to Nanjing University of Chinese Medicine, Zhangjiagang 215600, China; ^2^Department of Endocrinology, Zhangjiagang TCM Hospital Affiliated to Nanjing University of Chinese Medicine, Zhangjiagang 215600, China; ^3^Department of Orthopedics, Zhangjiagang TCM Hospital Affiliated to Nanjing University of Chinese Medicine, Zhangjiagang 215600, China; ^4^Department of Orthopedics, The First Affiliated Hospital of Soochow University, Suzhou 215006, China

## Abstract

Hyperprolactinemia is a common endocrine disease in women of reproductive age. Research has shown that patients with hyperprolactinemia often have decreased bone mineral density and an increased risk of fractures. However, there is still a lack of effective treatments. Paeoniflorin, one of the primary bioactive components in peony, is widely used in traditional Chinese medicine. Research has shown that paeoniflorin promotes osteoblast differentiation. However, whether paeoniflorin plays a role in hyperprolactinemia-induced osteoblastogenesis inhibition is not yet clear. In this study, we investigated the effect of paeoniflorin on prolactin (PRL)-mediated inhibition of osteoblast function. Our results showed that prolactin significantly reduced the expression of alkaline phosphatase (ALP), Osterix, and runt-related transcription factor 2 (RUNX2) in MC3T3-E1 cells cultured in an osteoblast differentiation medium, suggesting that prolactin inhibited osteoblast function. After treatment with paeoniflorin (PF), the expression of these osteoblast markers was upregulated. In addition, our findings proved that paeoniflorin increased the absorbance values of ALP-positive cells and the areas of alizarin red S (ARS) deposition compared to those in the prolactin group, suggesting that paeoniflorin reversed the PRL-induced reduction in osteoblast differentiation. The PRL-induced activation of nuclear factor kappa B (NF-*κ*B) was significantly reversed by paeoniflorin, indicating that paeoniflorin promoted osteoblast function by inhibiting the NF-*κ*B signaling pathway. In summary, these results showed that paeoniflorin alleviated the inhibitory effect of prolactin on osteoblastogenesis by suppressing the NF-*κ*B signaling pathway.

## 1. Introduction

Hyperprolactinemia (HPRL) is characterized by dysfunction of the hypothalamic-pituitary-reproductive axis and mainly refers to serum levels of PRL that exceed normal values (PRL > 25 ng/ml). The incidence rate of HPRL is 0.4% in the general population and as high as 9% to 17% in women with reproductive disorders. In premenopausal women, HPRL can lead to oligomenorrhea, amenorrhea, and anovulatory cycles of infertility, while in men, it leads to impotence and breast development [1]. Studies have shown that basal circulating PRL (7–10 ng/ml) is essential for maintaining normal bone growth and remodeling [2, 3]. High physiological levels of PRL during pregnancy (75–100 ng/ml) and prolonged lactation (200–350 ng/ml) can lead to transient osteopenia and a negative calcium balance [4–6]. Although lactation-induced osteopenia can be reversed after weaning, there have been reports of severe osteoporosis and an increased risk of fracture [7, 8]. Pathological conditions associated with chronic HPRL (up to 1000 ng/ml), such as prolactinoma and prolonged antipsychotic drug use, result in progressive osteopenia and osteoporosis [9–11]. Thus, the treatment of HPRL-induced bone loss is a subject requiring close clinical attention.

PRL-mediated suppression of estrogen synthesis has long been considered a mechanism by which HPRL induces bone loss [12, 13]. The bone may be the direct target of PRL; however, osteoblasts express transcripts for PRL receptors (PRLRs) [14, 15]. Since osteoclasts do not express PRLRs, HPRL-induced bone loss could be due to PRL-mediated inhibition of osteoblast function. Studies have shown that HPRL reduces the expression of the osteoblast gene *Runx2*, downregulates the mRNA of *osteoprotegerin (OPG)*, and increases the protein expression of the receptor activator for nuclear factor-*κ*B ligand (RANKL) [13, 16, 17]. Therefore, improving the function of osteoblasts may be an effective way to inhibit HPRL-induced bone loss. Currently, parathyroid hormone (PTH) is the main factor that promotes bone formation. However, prolonged administration of PTH can result in hypercalcemia [18]. In addition, the persistent role of PTH in promoting osteoblasts is also a problem to be addressed. Consequently, it is critical to identify new treatments for HPRL-induced osteoporosis. Traditional Chinese medicine is widely used to prevent and treat various diseases and is well-known for its affordability, mild side effects, and safety. This type of treatment is the current research direction of most scholars.

Paeoniflorin, a monoterpene glycoside, has the most abundant bioactive components in the Chinese herb peony, including Radix Paeoniae Rubra and Radix Paeoniae Alba. PF exhibits antioxidant [19–21], anti-inflammatory [20–22], antiapoptotic [21], antihyperlipidemic [23], and analgesic effects [24]. Ni et al. [25] discovered that PF could inhibit alveolar bone resorption in experimental periodontitis. Wang et al. [26] further confirmed that PF dose-dependently upregulated the expression of the transcription factor RUNX2 in primary osteoblasts and promoted osteoblast differentiation. However, it is not known whether PF has an effect on HPRL-induced osteoblastogenesis inhibition.

Prolactin, a hormone produced by the pituitary gland, has multiple physiological functions, including immunoregulation. The immune-modulatory activities of PRL may arise from increasing nuclear transcription factors such as IRF-1 and NF-kB, which play a pivotal role in many immune functions [27]. Ochoa-Amaya et al. [28] found that short-term hyperprolactinemia has proinflammatory effects. Olavarría et al. [29] also discovered the capacity of PRL to promote, through the NF-kB signaling pathway, the polarization of fish macrophages to a proinflammatory M1/classically activated phenotype characterized by the production of ROS and proinflammatory cytokines. However, it is not clear whether PRL plays a role in osteoblastogenesis inhibition through the NF-kB signaling pathway.

**Scheme 1:**
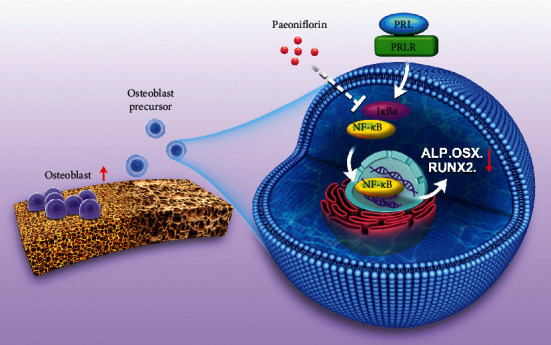
PF ameliorated HPRL-induced osteoblastogenesis inhibition by promoting osteoblast differentiation by inhibiting nuclear factor kappa B (NF-*κ*B) activity. PRL, prolactin; PRLR, prolactin receptor; ALP, alkaline phosphatase; OSX, Osterix; RUNX2, runt-related transcription factor 2; I*κ*B-*α*, inhibitor of kappa B alpha.

In our study, we established models of PRL-induced inhibition of osteoblast function in vitro to observe the effect of PF on PRL-induced inhibition of osteoblastogenesis. Our findings suggested that PF ameliorated HPRL-induced osteoblastogenesis inhibition by promoting osteoblast differentiation by inhibiting NF-*κ*B activity (Scheme 1). Therefore, drugs containing PF as the main active ingredient may be an effective way to treat HPRL-induced osteoporosis.

## 2. Results

### 2.1. Prolactin Dose-dependently Inhibited Osteoblast Differentiation In Vitro

**Figure 1:**
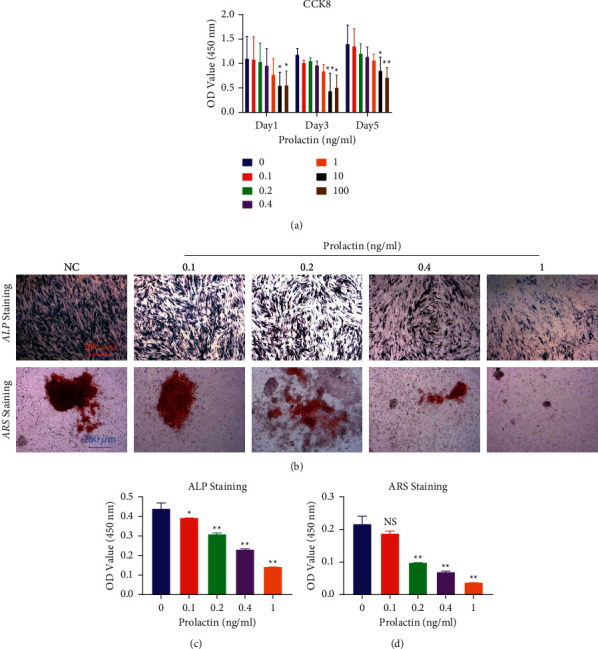
Prolactin inhibited osteoblast differentiation in vitro. (a) A CCK-8 assay was performed to evaluate cell viability. (b) Representative images of ALP and ARS staining. (c) The ALP-positive cells in each group were quantified. (d) Quantification of ARS-stained cells and mineral nodules. n = 3; scale bar = 200 *μ*m; ^#^*P* < 0.05, ^##^*P* < 0.01, ^#^ vs the 0 ng/ml group; NS: not statistically significant, ^*∗*^*P* < 0.05, ^*∗∗*^*P* < 0.01, ^*∗*^ vs the NC group. ALP, alkaline phosphatase; ARS, alizarin red S.

The cell counting kit-8 (CCK-8) assay results indicated that cell viability was unaffected by treatment with PRL at concentrations below 1 ng/ml for 1, 3, and 5 days (Figure 1(a)). To examine the role of PRL in osteoblast differentiation, ALP and ARS staining were used. As shown in Figures 1(b)-1(c), visualization of the cells under an optical microscope revealed that the size of the ALP-positive area decreased as the PRL concentration increased, while analysis of ALP-positive cells revealed that PRL inhibited the differentiation of osteoblasts at all concentrations (0.1–1 ng/ml). ARS staining of mineralization showed that PRL reduced mineralization in vitro (Figure 1(b)). Quantification of the mineral nodules revealed that PRL suppressed mineralization associated with osteogenesis in a dose-dependent manner (Figure 1(d)).

**Figure 2:**
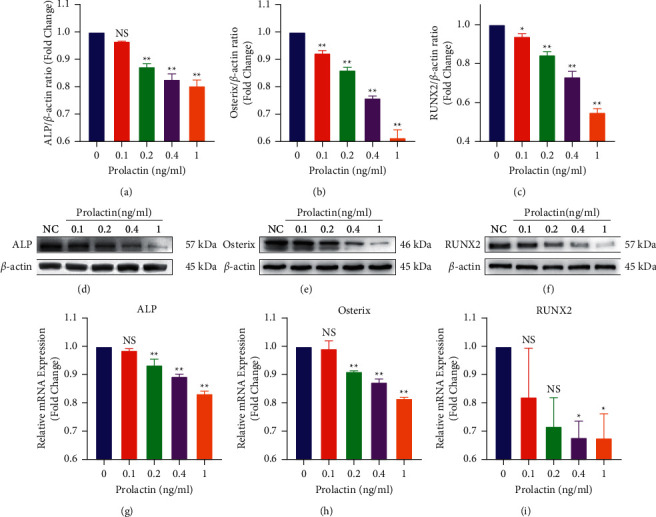
Prolactin inhibited the expression of osteoblast-related proteins and genes. (a–c) Quantification of the osteoblast-related proteins ALP, Osterix, and RUNX2. (d–f) Cell lysates were subjected to WB analysis with antibodies against these proteins. (g–i) Quantification of the mRNA expression of *ALP*, *Osterix*, and *RUNX2*. *n* = 3; NS: not statistically significant, ^*∗*^*P* < 0.05, ^*∗∗*^*P* < 0.01, ^*∗*^ vs the NC group.

Western blot (WB) analysis and quantitative real-time polymerase chain reaction (qRT-PCR) were used to assess whether PRL suppressed osteoblast-related protein and gene expression, respectively. MC3T3-E1 cells were cultured in an osteogenic induction medium and treated with various concentrations of PRL (0.1, 0.2, 0.4, and 1 ng/ml). The WB results verified that the expression of proteins, including ALP, Osterix, and RUNX2, was markedly downregulated by PRL (*P* < 0.05) (Figures 2(a)–2(f)). Moreover, the same trend was observed in the mRNA expression levels of *ALP*, *Osterix*, and *RUNX2* (Figures 2(g)–2(j)). These results demonstrated that PRL suppressed osteoblast differentiation in a dose-dependent manner.

### 2.2. Paeoniflorin Ameliorated Hyperprolactinemia-induced Inhibition of Osteoblastogenesis

**Figure 3:**
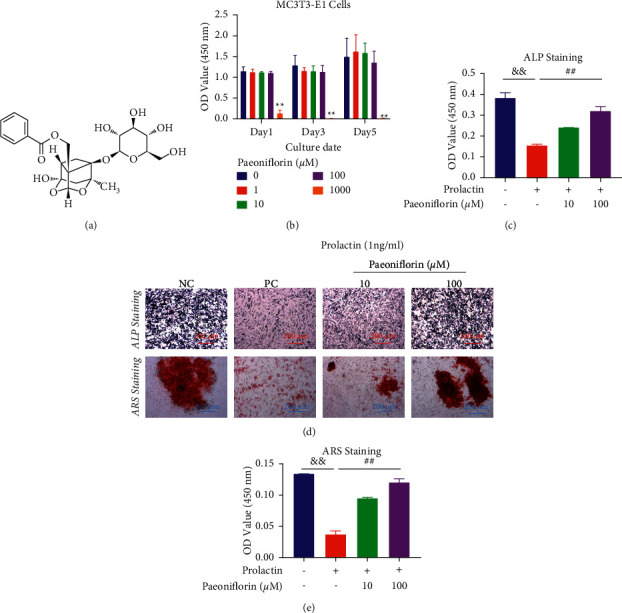
Paeoniflorin ameliorated HPRL-induced osteoblast differentiation inhibition in vitro. (a) The structure of PF. (b) A CCK-8 assay was performed to evaluate cell viability. (c) Representative images of ALP and ARS staining. (d) The ALP-positive cells in each group were quantified. (e) Quantification of ARS-stained cells and mineral nodules. *n* = 3; scale bar = 200 *μ*m; ^*∗∗*^*P* < 0.01, ^*∗*^ vs the 0 ng/ml group; ^&&^*P* < 0.01, ^&^vs the NC group; ^##^*P* < 0.01, ^#^ vs the PC group.

To analyze the effect of PF on osteoblast viability, a CCK-8 assay was performed. As shown in Figure 3(b), PF had no toxic effects on MC3T3-E1 cells at concentrations below 100 *μ*M for 1, 3, and 5 days.

MC3T3-E1 cells were incubated in an osteogenic induction medium and treated with 1 ng/ml PRL and different concentrations of PF (10 and 100 *µ*M) for 2 and 3 weeks. ALP and ARS staining showed that PF dose-dependently reversed the PRL-induced reduction in osteoblast differentiation (Figure 3(c)). The quantification of ALP-positive cells is shown in Figure 3(d), and the absorbance values in the PF groups that were treated with 10 and 100 µM were 0.2420 ± 0.0017 nm and 0.3210 ± 0.0245 nm, respectively. Compared with 0.1557 ± 0.0091 nm in the PRL group, these values were statistically significant. ARS-positive areas were quantified, and the absorbance value in the PRL group was 0.03733 ± 0.00643 nm. In comparison, the value increased to 0.09533 ± 0.00208 nm and 0.1207 ± 0.0067 nm after treatment with 10 and 100 µM PF, respectively (Figure 3(e)).

**Figure 4:**
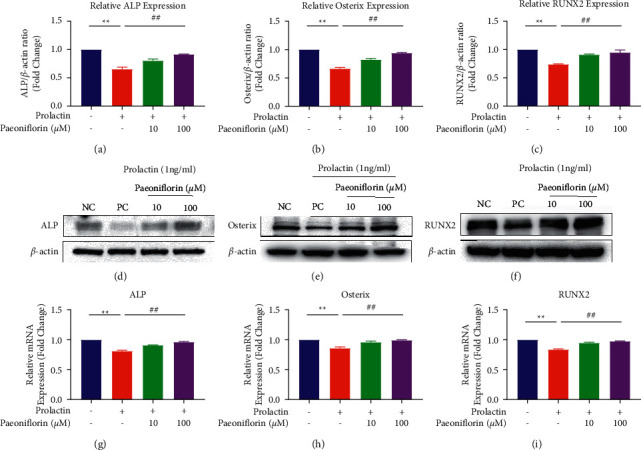
Paeoniflorin rescued hyperprolactinemia-induced inhibition of osteoblast-related protein and gene expression. (a–c) Quantification of the osteoblast-related proteins ALP, Osterix, and RUNX2. (d–f) Cell lysates were subjected to WB analysis with antibodies against these proteins. (g–i) Quantification of the mRNA expression of *ALP*, *Osterix*, and *RUNX2*. *n* = 3; ^*∗∗*^*P* < 0.01, ^*∗*^vs the NC group; ^##^*P* < 0.01, ^#^vs the PC group.

In addition, we also observed that PF significantly increased the protein expression of ALP, Osterix, and RUNX2 compared with that in the PRL group (*P* < 0.01). After treatment with 10 and 100 µM PF, the expression of these proteins was upregulated nearly 1.2-fold compared with that in the positive control group (Figures 4(a)–4(f)). Moreover, the mRNA expression levels of these genes were significantly increased in a concentration-dependent manner (*ALP*: 0.9098 ± 0.0061, 0.9616 ± 0.0127, and 0.8129 ± 0.0123; *Osterix*: 0.9598 ± 0.0197, 0.9945 ± 0.0056, and 0.8612 ± 0.0189; and *RUNX2*: 0.9483 ± 0.0112, 0.9767 ± 0.0054, and 0.8386 ± 0.0098) (Figures 4(g)–4(i)).

### 2.3. Paeoniflorin and the NF-*κ*B Signaling Pathway

**Figure 5:**
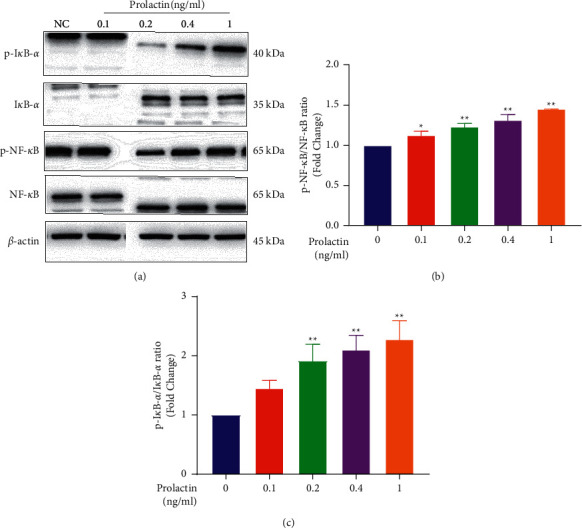
Prolactin activated the NF-*κ*B signaling pathway during osteoblastogenesis. (a) Cell lysates were subjected to WB analysis with antibodies against phospho-I*κ*B-*α*, I*κ*B-*α*, phospho-NF-*κ*B, and NF-*κ*B. (b and c) The ratios of p-I*κ*B-*α*/I*κ*B-*α* and p-NF-*κ*B/NF-*κ*B. *n* = 3, NS: not statistically significant, ^*∗*^*P* < 0.05, ^*∗∗*^*P* < 0.01, ^*∗*^vs the NC group.

The NF-*κ*B signaling pathway is an essential molecular response to various types of cellular stress. Xu et al. [27] and Zheng et al. [28] confirmed that this signaling pathway plays a crucial role in the differentiation of osteoblasts. PRL has been shown to affect the NF-*κ*B signaling pathway [29, 30]. Thus, we further investigated whether PRL affected osteoblast function via the NF-*κ*B signaling pathway. WB analysis showed that the phosphorylation of I*κ*B-*α* and NF-*κ*B was dose-dependently enhanced by PRL (s Figure 5(a)–5(c)), suggesting that PRL suppressed osteoblastogenesis by activating the NF-*κ*B signaling pathway.

**Figure 6:**
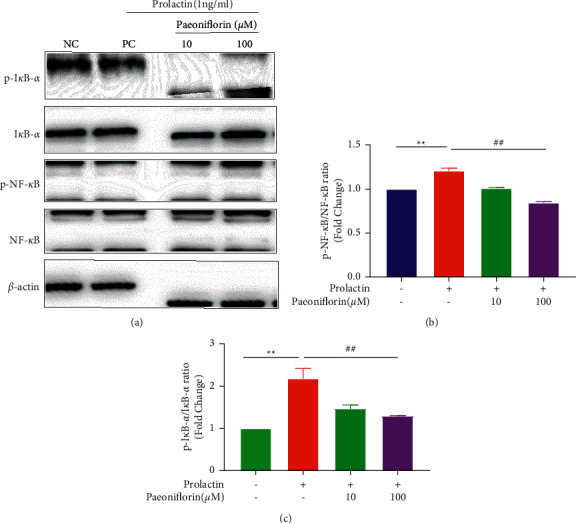
Paeoniflorin suppressed HPRL-induced NF-*κ*B signaling pathway activation during osteoblastogenesis. (a) Cell lysates were subjected to WB analysis with antibodies against phospho-I*κ*B-*α*, I*κ*B-*α*, phospho-NF-*κ*B, and NF-*κ*B. (b and c) The ratios of p-I*κ*B-*α*/I*κ*B-*α* and p-NF-*κ*B/NF-*κ*B. *n* = 3, ^*∗∗*^*P* < 0.01, ^*∗*^vs the NC group; ^##^*P* < 0.01, ^#^vs the PC group.

Our findings indicated that PF could promote osteoblast differentiation. Considering that PRL inhibited osteoblast function through the NF-*κ*B signaling pathway, we further explored whether PF rescued PRL-induced inhibition of osteoblast differentiation via this signaling pathway. MC3T3-E1 cells were incubated in the osteogenic induction medium and treated with 1 ng/ml PRL and different concentrations of PF (10 and 100 µM) for 3 days, and then, WB analysis was performed. As shown Figures 6(a)–6(c), p-NF-*κ*B and p-I*κ*B-*α* were dose-dependently suppressed by PF in the presence of PRL.

## 3. Discussion

HPRL is a common cause of female reproductive disorders. Both physiological and pathological HPRL can lead to osteoporosis, which seriously threatens bone health in women over time [4–7, 31–34]. Currently, there is still a lack of safe and effective treatments for HPRL-induced osteoporosis. Osteoblasts are directly involved in bone formation and play a crucial role in HPRL-induced bone loss. Thus, osteoblasts are a therapeutic target in HPRL-induced osteoporosis. It has been well documented that osteoblast functions are suppressed in the presence of PRL [15, 16]. Our results also confirmed that PRL inhibited osteoblast function at concentrations of 0.1 to 1 ng/ml. It has been reported that PRL decreases the protein or mRNA expression of osteogenic markers, including *ALP*, *OCN*, *OPN*, and *RUNX2*, to inhibit osteoblastogenesis [14, 17]. Our study was consistent with these previous reports that the expressions of proteins and mRNA, including *ALP*, *Osterix* and *RUNX2*, were markedly downregulated by PRL, which demonstrated that PRL suppressed osteoblast differentiation. All these results proved that increasing osteoblast function can rescue PRL-induced osteoblastogenesis inhibition.

PF is a small molecule compound with strong adaptability and many targets. It was reported that PF had protective effects on advanced oxidation protein product (AOPP)-induced oxidative injury in human umbilical vein endothelial cells (HUVECs) [35]. In addition, Chen et al. [36] showed that PF ameliorated *α*-naphthylisothiocyanate (ANIT)-induced cholestasis in rats. Wang et al. [37] showed that PF could reduce bone loss induced by hyperlipidemia. In this study, we established models of osteoblast differentiation inhibition induced by PRL in vitro. We found that PF treatment can promote osteoblast function in the presence of PRL. This result is consistent with a previous study showing that PF facilitates osteoblastogenesis and thus reduces ovariectomy-induced osteoporosis in ovariectomized mice [26]. Paeoniflorin, as a water-soluble monoterpene glycoside [38], cannot penetrate directly through the phospholipid bilayer. Therefore, we speculate that PF is transported across the cell membrane through passive transport; however, the specific mechanism needs to be further studied.

NF-*κ*B is a family of dimeric transcription factors that is fundamental to cellular differentiation, proliferation, and survival in almost all multicellular organisms. Interactions with the inhibitor protein I*κ*B controls NF-*κ*B. NF-*κ*B can be activated by signal-induced degradation of the I*κ*B-*α* protein. As I*κ*B-*α* degrades, NF-*κ*B is activated and translocates into the nucleus, where it binds to target DNA sites and activates the transcription of genes encoding proteins that control cell growth. Previous studies have shown that PRL affects the NF-*κ*B signaling pathway [39, 40]. Similarly, our findings revealed that PRL suppressed osteoblastogenesis by activating the NF-*κ*B signaling pathway. Numerous studies have proven that the NF-*κ*B signaling pathway is important in regulating osteogenesis [30, 41, 42]. Wang et al. [43] showed that scutellarin promoted osteoblast proliferation and function via the NF-*κ*B/p65 signaling pathway. Liu et al. [44] proved that pilose antler peptide enhanced osteoblast differentiation by regulating the NF-kB pathway. Similarly, we found that PF can inhibit the phosphorylation of IkB*α* and p65 in osteoblast differentiation inhibition induced by PRL, which demonstrated that PF alleviated PRL-induced inhibition of osteoblast function by suppressing the NF-*κ*B signaling pathway.

In summary, our study demonstrated that PF could promote osteoblast differentiation in the presence of PRL by suppressing the NF-*κ*B signaling pathway and reduce PRL-induced osteoblastogenesis inhibition. Therefore, PF may be a potential treatment for bone loss induced by HPRL.

## 4. Materials and Methods

### 4.1. Materials

PF (C_23_H_28_O_11_, MW: 480.468, high-performance liquid chromatography purity ≥98%, P0038, Sigma-Aldrich, Shanghai, China) and human pituitary PRL (purity ≥98%, 869039, Sigma-Aldrich, Shanghai, China) were purchased commercially.

### 4.2. Cell Viability Assay

To examine the cytotoxicity of PRL or PF on MC3T3-E1 cells, a cell counting kit-8 (CCK-8) viability assay (CK04, Dojindo, Shanghai, China) was performed. MC3T3-E1 cells were plated in 96-well plates (5 × 10^4^ cells/well) for 12 hours to ensure cell adhesion. After 1, 3, and 5 days of incubation with PRL (0, 0.1, 0.2, 0.4, 1, 10, or 100 ng/ml) or PF (0, 1, 10, 100, or 1000 *μ*M), 10-*μ*l CCK-8 was added to each well, and the cells were incubated at 37°C and 5% CO_2_ for an additional 1 hour. The absorbance of each well was measured at 450 nm by a microplate reader (ELx800, Bio-Tek, USA).

### 4.3. Alkaline Phosphatase (ALP) Staining

MC3T3-E1 cells were cultured in 24-well plates with the osteogenic induction medium and treated with or without PRL (0, 0.1, 0.2, 0.4, or 1 ng/ml) and PF (10 *μ*M or 100 *μ*M) for 2 weeks. Then, the cells were fixed with 4% formaldehyde, rinsed three times with PBS (C0221A, Beyotime Biotechnology, Shanghai, China), and treated according to the protocol of the BCIP/NBT alkaline phosphatase color development kit (C3206, Beyotime Biotechnology, Shanghai, China). After an additional wash, the ALP-positive cells were photographed via light microscopy and observed at 450 nm in a microplate reader.

### 4.4. Alizarin Red S (ARS) Staining and Mineralization Assay

MC3T3-E1 cells were cultured in 24-well plates with the osteogenic induction medium and treated with or without PRL (0, 0.1, 0.2, 0.4, or 1 ng/ml) and PF (10 *μ*M or 100 *μ*M) for 3 weeks. Then, the cells were fixed with 4% formaldehyde, rinsed three times with PBS, and treated with 0.2% ARS solution (G1450, Solarbio, Beijing, China). After an additional wash, the stained cells and mineral nodules were photographed and observed at 450 nm in a microplate reader.

### 4.5. Western Blot (WB) Analysis

MC3T3-E1 cells were seeded at 5 × 10^5^ cells/well in 6-well plates, incubated with the osteogenic induction medium and treated with or without PRL (1 ng/ml) and PF (10 *μ*M or 100 *μ*M) for the indicated times. Then, the cells were lysed with a RIPA lysis buffer (P0013 B, Beyotime Biotechnology, Shanghai, China) containing a protease inhibitor cocktail (P1010, Beyotime Biotechnology, Shanghai, China). After protein quantification, 20-*μ*g sample was loaded onto gels, separated by 4–20% SDS-PAGE (P0468 M, Beyotime Biotechnology, Shanghai, China), and transferred onto PVDF membranes (IPVH00010, Millipore). Beta-actin was detected separately when the molecular weight of the target protein, including ALP, RUNX2, p-NF-*κ*B, and NF-*κ*B, was largely different from it. When target proteins such as Osterix, p-I*κ*B-*α*, and I*κ*B-*α* with molecular weight close to that of *β*-actin were detected, *β*-actin was detected after stripping with a Stripping buffer (P0025, Beyotime Biotechnology, Shanghai, China) and reprobing. Next, the membranes were blocked in a QuickBlock Blocking Buffer for Western blot (P0252, BeyotimeBiotechnology, Shanghai, China) at room temperature for 15 minutes and incubated with rabbit primary antibodies at 4°C overnight. The antibodies used were as follows: ALP (1 : 1000, ab65834, Abcam, UK), Osterix (1 : 1000, ab209484, Abcam, UK), RUNX2 (1 : 1000, ab23981, Abcam, UK), I*κ*B-*α* (1 : 1000, ab32518, Abcam, UK), NF-*κ*B (1 : 2000, ab16502, Abcam, UK), *β*-actin (1 : 1000, ab8227, Abcam, UK), p-I*κ*B-*α* (1 : 1000, 2859, Cell Signaling Technology, USA), and p-NF-*κ*B (1 : 1000, 3031, Cell Signaling Technology, USA). After being washed with TBST (CW0043S, CWBiotech) for 30 minutes, the membranes were incubated with secondary antibodies (1 : 1000, 5571, Cell Signaling Technology, USA) for 1 hour at room temperature. Finally, the proteins were analyzed with a chemiluminescent HRP substrate (WBKLS0500, Millipore Corporation). The protein bands were visualized by ImageQuant LAS 500 (Ge Healthcare Bio-Sciences AB, Sweden), and the gray values were analyzed by ImageJ software (NIH, USA) and normalized to *β*-actin. The experiments were repeated at least 3 times.

### 4.6. RNA Extraction and Quantitative Real-time PCR

#### 4.6.1. RNA Extraction and Reverse Transcription

MC3T3-E1 cells were cultured in a 6-well plate in the osteogenic induction medium with or without PRL (1 ng/ml) and PF (10 *μ*M or 100 *μ*M) for 2 days. The cells were washed with PBS for 3 times in ice and lysed with 1 ml of TRIzol reagent (15596018, Ambion, USA). After complete dissolving at room temperature, the samples were removed to a new 1.5 ml microcentrifuge tube. Next, 200-*μ*l chloroform was added and shaked vigorously, and then, the mixture was incubated at room temperature for 10 min. Later, the tube was centrifuged at 12000 × *g* for 15 min, then three distinct phases were observed in the mixture. 400 *μ*l of the upper colorless phase was transferred into 400-*μ*l cold isopropanol in a separate tube. The tube was incubated at room temperature for 10 min after gently mixing by inverting the closed tube several times. The tube was centrifuged at 12000 × *g* for 15 min, and then, isopropanol was discarded. RNA was precipitated as a pellet in the bottom of the tube. 1-ml 75% cold ethanol was added into the tube. Afterwards, the tube was centrifuged at 7500 × *g* for 5 min at 4°C, and then, the ethanol was discarded. After that, RNA was dissolved in 20-*μ*l RNase-free water, and the concentration was detected by a NanoDrop™ One/OneC Microvolume UV-Vis Spectrophotometer (Thermo Fisher Scientific, USA). The ratio of the absorbance at 260 and 280 nm (A260/280) was used to assess RNA purity. The A260/A280 ratio at 1.8–2.0 is acceptable. Then, reverse transcription was performed to convert the RNA to cDNA. First, cDNA was synthesized using the Prime Script RT Master Mix kit (G492, Abm, Canada). 4 *μ*l of Prime Script RT Master Mix, dH2O, and 1 *μ*g of UV-quantified RNA were mixed. The reactions were incubated at 25°C for 10 min, 42°C for 15 min, 85°C for 5 min, and 4°C for infinite time by a thermal cycler (Applied Biosystems, USA). Samples were transferred to ice or at −20°C for long-term storage.

#### 4.6.2. Real Time RT-PCR

Set up a real-time PCR master mix (total volume 18 *μ*l) as follows: 10 *μ*l of SYBR Green Master Mix, 7 *μ*l RNase-free water, 0.5 *μ*l of sense primer, and 0.5 *μ*l of antisense primer. Combine 18 *μ*l of real-time PCR master mix (see above) with 1 *μ*l sample of cDNA and 1 *μ*l dH_2_O in a 200 *μ*l PCR tube. The reactions were performed in 96-well plates in a LightCycler 96 instrument (Roche Diagnostics GmbH, Germany) with SYBR Green Master Mix (4364346, Applied Biosystems, USA) following the parameters: 94°C for 10 minutes, followed by 40 cycles of 95°C for 15 seconds and 60°C for 60 seconds. A “no-template” control well consisting of all the components of the real-time PCR master mix, except the sample cDNA, was used to control for extraneous DNA contamination. *GAPDH* was used to normalize the expression of target genes. The sequences for the forward and reverse primers used in this study were as follows: *ALP* (forward: 5′-CCAACTCTTTTGTGCCAGAGA-3′, reverse: 5′-GGCTACATTGGTGTTGAGCTTTT-3′), *Osterix* (forward: 5′-GGAAAGGAGGCACAAAGAAGC-3′, reverse: 5′-CCCCTTAGGCACTAGGAGC-3′), *RUNX2* (forward: 5′-GACTGTGGTTACCGTCATGGC-3′, reverse: 5′-ACTTGGTTTTTCATAACAGCGGA-3′), and *GAPDH* (forward: 5′-AGGTCGGTGTGAACGGATTTG-3′, reverse: 5′-GGGGTCGTTGATGGCAACA-3′). Data acquisition is performed automatically by the PCR equipment software. For each sample, we calculated the average Ct (Ct_av_) of the three technical replicates for both the target genes and the endogenous reference gene as follows: Ct_av_= (Ct_1_ + Ct_2_ + Ct_3_)/3; Ct_1_, Ct_2_, and Ct_3_ are the average values of the three technical replicates. The relative amount of target genes was calculated for each sample using the ΔCt method [45] as follows: ΔCt_av_ = [Ct_av_(end)−Ct_av_(target gene)], where end is the endogenous gene used as reference. The fold change (FC) of the target genes DNA amount between different samples were calculated as follows: FC = 2^−ΔCtav^. We measured the relative amount of target genes DNA present in a sample compared to that of the control group.

### 4.7. Statistical Analysis

The data from each experiment are presented as mean ± standard error of the mean (SEM) and were analyzed by Prism for Windows (version 8.0, GraphPad Software Inc., USA). Multiple comparisons were performed by one-way analysis of variance (ANOVA) with Newman–Keuls posttests. *P*-values less than 0.05 were considered to be statistically significant.

## Data Availability

The data used to support the findings of this study are available from the corresponding author upon request.
